# Is Yessotoxin the Main Phycotoxin in Croatian Waters?

**DOI:** 10.3390/md8030460

**Published:** 2010-03-05

**Authors:** Živana Ninčević Gladan, Ivana Ujević, Anna Milandri, Ivona Marasović, Alfiero Ceredi, Silvia Pigozzi, Jasna Arapov, Sanda Skejić, Stjepan Orhanović, Igor Isajlović

**Affiliations:** 1 Institute of Oceanography and Fisheries, Šet. I. Meštrovića 63, 21000 Split, Croatia; E-Mails: ujevic@izor.hr (I.U.); marasovic@izor.hr (I.M.); arapov@izor.hr (J.A.); sanda@izor.hr (S.S.); igor@izor.hr (I.I.); 2 Fondazione Centro Ricerche Marine National Reference Laboratory on Marine Biotoxins, 47042 Cesenatico, Italy; E-Mails: anna.milandri@centroricerchemarine.it (A.M.); alfiero.ceredi@centroricerchemarine.it (A.C.); silvia.pigozzi@centroricerchemarine.it (S.P.); 3 Faculty of Science, University of Split, Teslina 12, 21000 Split, Croatia; E-Mail: Stjepan.Orhanovic@pmfst.hr (S.O.)

**Keywords:** yessotoxin, okadaic acid, Lingulodinium polyedrum, Dinophysis fortii, Adriatic Sea

## Abstract

With the aim of investigating whether yessotoxin (YTX) is responsible for diarrhetic shellfish poisoning (DSP) events in Croatian waters, three different methods were combined: a modified mouse bioassay (MBA) that discriminates YTX from other DSP toxins, the enzyme-linked immunosorbent assay method (ELISA) and liquid chromatography-mass spectrometry (LC-MS/MS). Among 453 samples of mussels and seawater analyzed in 2007, 10 samples were DSP positive. Results obtained by the modified MBA method revealed that most of the samples were positive for YTX, with the exception of samples from Lim Bay (LB 1) The ELISA method also identified the presence of YTX in these samples. DSP toxin profiles showed the presence of okadaic acid (OA) in three, and YTX in four out of nine samples that were analyzed by LC-MS/MS. The phytoplankton community structure pattern revealed *Lingulodinium polyedrum* (Stein) Dodge, which was present in the water prior to and/or during toxicity events at low concentrations (80 to 1440 cells L^−1^), as a potential YTX producing species. It is proposed that *L. polyedrum* cells accumulated in mussels and the subsequently observed toxicity may be related to metabolism after ingestion, resulting in carboxy YTX as the major analog in the mussel.

## 1. Introduction

Microscopic planktonic algae are critical food for filter-feeding shellfish including oysters, mussels, scallops and clams. In most cases, intensive proliferation of phytoplankton cells (so-called algal blooms; up to millions of cells per liter) is beneficial for aquaculture and fishery harvesting. However, in some situations, algal blooms can cause severe damage to aquaculture and can have an adverse impact on human health. Among the 5000 species of extant marine phytoplankton according to Sournia *et al.* [1], there are about 80 species that have the capacity to produce potent toxins [2], which can, through the food web, have a negative impact on human health and cause a variety of gastrointestinal and neurological illnesses. There are several types of toxicity, which are divided by the symptoms they cause in sea mammals and humans. In Croatian waters, only toxins associated with Diarrheic Shellfish Poisoning (DSP) have been identified to date in concentrations that can impact on human health [3,4].

The first record was during the summer of 1989 on the north-west coast of the Adriatic Sea when the presence of dinoflagellates genera *Dinophysis* and *Prorocentrum* resulted in shellfish intoxication with Diarrheic Shellfish Poisoning (DSP) [5]. Subsequent DSP episodes in Croatian waters have been reported. The occurrence of DSP toxicity in the middle Adriatic was first registered in the summer of 1993 in Kaštela Bay [6] and has been repeatedly observed [7,8]. The National monitoring program of shellfish breeding areas has revealed that most of the DSP events have occurred in the northern Adriatic [3,9].

The DSP toxins are all heat-stable polyether and lipophilic compounds that have been isolated from various species of shellfish and dinoflagellates [10]. Originally they were comprised of three groups of polyether toxins because they often co-occur and their toxins are coextracted in the same lipophilic fraction from plankton cells and shellfish. The first group, comprising acidic toxins, includes okadaic acid (OA) and its derivatives named dinophysistoxins (DTXs). The second group, comprising neutral toxins, consists of polyether-lactones of the pectenotoxin group (PTXs), while the third group includes a sulfated compound called yessotoxin (YTX), a brevetoxin-type polyether, and its derivative 45-hydroxyyessotoxin (45-OH-YTX) [10,11]. Nowadays, it is known that these three groups of toxins have different biological effects. YTXs are nondiarrheagenic, and compared to OA show a much lower potency for the inhibition of protein phosphatase 2A. For this reason, it has been proposed that YTXs should not be included in the list of DSP toxins.

DSP toxin profiles in Croatian waters have shown that OA was only occasionally the main toxin causing DSP toxicity events [3,9]. In most cases where DSP toxicity in shellfish was detected by mouse bioassay, OA was not present in sufficient concentrations to account for the recorded toxicity [6–8]. These findings imply that an unidentified DSP compound might have contributed to the toxic effect. Since YTX occurrence in shellfish from the middle Adriatic has been reported [12] as well as the presence of the dinoflagelate *Lingulodinium polyedrum* that is known to produce yessotoxin, we hypothesized that YTX could be the toxin responsible for most of the DSP toxicity events in Croatian waters. With the aim of investigating whether YTX is responsible for most of the DSP events in Croatian waters we combined a modified Yasumoto’s method, which allows us to extract YTX among other DSP toxins [13,14] with LC-MS/MS analysis of these lipophilic toxins.

## 2. Results and Discussion

Among 453 mussels and seawater samples analyzed in 2007, 10 samples were DSP positive (Tables 1 and 2). Investigations have suggested the presence of DSP toxins other than OA in shellfish from Croatian waters [3,6–8]. Results obtained in the period when the method that discriminated YTX from others DSP toxins [14] was used revealed that most of the samples were positive for YTX, except samples from Lim Bay (LB 1) (Table 2). The ELISA method identified the presence of YTXs in mussels (Tables 1 and 2). The DSP toxin profiles showed the presence of OA in three samples and YTXs in four samples (Table 3), out of the nine samples that were analyzed by LC-MS/MS. In two of the samples that tested positive for YTX using the modified Yasumoto’s method, this toxin was not found and could be due to the presence of YTX analogs, including metabolites in the shellfish, which were not analyzed for using the LC-MS/MS method.

At the station in Medulin Bay (MB), modified Yasumoto’s method revealed the presence of YTX and this was confirmed by a LC-MS/MS toxin profile that showed a high concentration of YTX analogs. Hydroxylated and carboxylated YTX derivatives are largely a result of the metabolism of YTX in shellfish after ingestion [15]. The most abundant of the YTXs in shellfish from station MB was carboxy-homo YTX, contributing to 64% of the total identified YTXs. According to Ciminiello *et al.* [16], the profiles of YTXs in *Mytilus galloprovincialis* collected at different times in the Adriatic Sea revealed YTX and homo YTX as the most abundant. High abundances of carboxy YTX have been reported in *Mytilus edulis* collected in Flødevigen Bay (Norway) when the total levels of YTXs were decreasing and were close to the lowest levels for that year, while YTX and 45-hydroxy YTX were predominant during and shortly after a bloom of *Protoceratium reticulatum* [16]. These findings imply that after absorption from the alga, YTX was rapidly oxidized to 45-hydroxy YTX and more slowly to carboxy YTX. In the remaining three samples from stations SB3, IP3 and IP2, YTXs were determined in much lower concentrations and mostly in the form of YTX and homo YTX.

Results obtained with these two methods confirmed that YTX could be the major toxin in shellfish from Croatian waters and the cause of positive DSP mouse bioassays. The response of the modified Yasumoto’s MBA method was higher for YTXs than the measured values of known YTX analogs by LC-MS and could be a result of the presence of other YTXs analogs. Since its discovery, close to 90 other analogs have been identified [17–20]. The response from the ELISA method was more sensitive than from LC-MS/MS. Indeed the greater sensitivity of the ELISA method and its greater response to YTXs in both mussels and phytoplankton has already been reported [15,21,22]. Those authors attributed this higher sensitivity to antibodies in ELISA to other YTX analogs whereas the LC-MS method only quantifies those YTX congeners that have standards associated with them.

**Figure 1:**
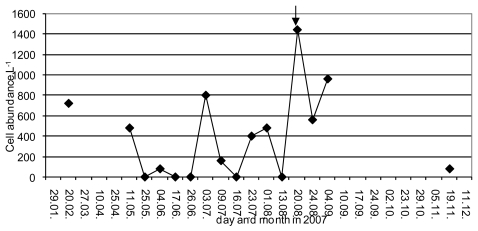
Abundance of *L. polyedrum* at station MB1. The arrow indicates when YTX was determined in mussels.

The biological origins of YTX are from the dinoflagellates *Protoceratium reticulatum* (Claparhde and Lachmann) Buetschli, Lingulodinium polyedrum (Stein) Dodge, and Gonyaulax spinifera (Claparede and Lachmann) Diesing. The phytoplankton community structure pattern revealed *L. polyedrum* as a potential YTX-producing species. At station MB1, where the MBA test revealed the presence of YTX, and this was confirmed by LC-MS/MS analyses, *L polyedrum* was present in the phytoplankton community (Figure 1). YTX presence in mussel was recorded in non bloom conditions, although the continuous presence of *L. polyedrum* in abundances from 80 to 1440 cells L^−1^ four months prior to toxicity events was recorded. It is possible that *L. polyedrum* cells accumulated in mussels until they manifested as a toxicity event, and may be ascribed to carboxy YTX as the major analog in the mussel, due to metabolism after ingestion. In support of this are findings of Aasen *et al*. [21], who noted that after absorption from the algae YTX was rapidly oxidized to 45-hydroxy YTX and more slowly to carboxy YTX by blue mussel. The depuration rate for carboxy YTX was considerably slower than for YTX or 45-hydroxy YTX [21]. From seawater samples collected at four other stations where YTXs were recorded in mussels, *L. polyedrum* was also present in the phytoplankton assemblages. At station IP3 the abundance of *L. polyedrum* in May was up to 12,100 cells m^−2^. At station MSB4, *L. polyedrum* abundance ranged from 1,100 to 2200 cells m^−2^ in the period from the end of the August to the first half of September when MBA for YTX was positive. In addition, at this station in September, *G. spinifera* had been recorded in abundance of 1100 cells m^−2^. The YTXs profile in the producing organisms revealed that *G. spinifera* cultures produce unspecified YTXs identified by ELISA analysis [23], which could explain why YTX at this station was not determined by LC-MS/MS while MBA [13] and ELISA detected it. At station IP2, the first occurrence of *L. polyedrum* was in May with an abundance of 30,000 cells m^−2^ and again its appearance was recorded during shellfish toxicity at an abundance of 4400 cells m^−2^. *L. polyedrum* is a bloom forming species, which can produce intensive blooms with more than a million cells per liter, although for the cases reported here, relatively low concentrations of this species were noted during the entire investigation period. Remarkable amounts of YTXs associated with low concentrations of YTX producers in the Adriatic Sea [24] have been previously reported and attributed to the toxins’ accumulation.

At stations where OA was determined as the major toxin in shellfish, *Dinophysis* species were abundant in the phytoplankton community. In Mali Ston Bay at station MSB5, a potential toxin producer was *D. caudata*, which was present in the community from June to December, in an abundance ranging from 3300 to 75,480 cells m^−2^. In Lim Bay at station LB1, shellfish toxicity caused by OA presence occurred after an intensive bloom of *D. fortii* with an abundance of 31,080 cells L^−1^.

Knowledge of the toxicity of the various YTXs to humans is very limited and, although YTX is toxic by intraperitoneal exposure in the mouse bioassay (0.1–0.8 mg/kg), it appears to be of low toxicity when administered orally [25,26]. Comparison of results obtained by three different methods (MBA, ELISA, LC-MS/MS) showed good agreement, although the ELISA method appears to be the most sensitive. Due to the high sensitivity of ELISA and the relatively low toxicity of YTXs for humans when taken orally, further research is needed to determine regulatory levels when ELISA is employed, with the goal of protecting public health while minimizing the risk of unnecessary closures of shell fisheries.

## 3. Experimental Section

### 3.1. Sampling

**Figure 2:**
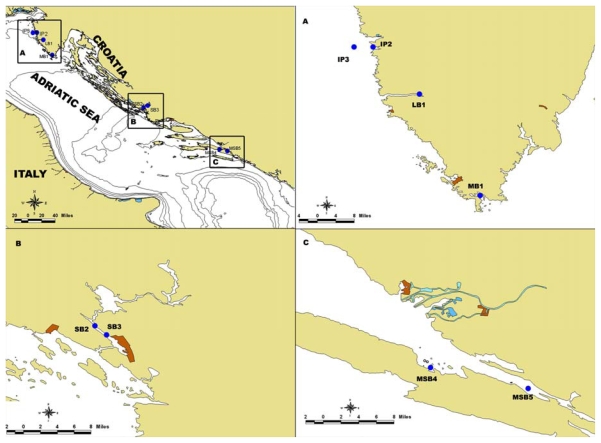
Investigated area with sampling stations. in Northern (A), Middle (B) and Southern (C) Adriatic Sea.

In the frame of the National Monitoring Programme of Shellfish Farms, both seawater and shellfish sampling were carried out monthly in the period from November to March and weekly in the period from April to October. In 2007, 453 samples, collected at 19 stations including the Istrian area, Šibenik Bay and Mali Ston Bay, were analyzed. In this paper mouse bio-assay positive DSP results obtained in 2007 were analyzed. Stations where positive results were recorded are shown in Figure 2.

### 3.2. Mouse Bio-assay (MBA)

Analyses of DSP toxicity by the mouse bio-assay were performed according to Yasumoto [13]. In the period from 20 August to 08 November 2007 a modified method [14], which allowed the differentiation of YTX positive results from other DSP positive results, was used.

#### 3.2.1. Sample Preparation

Samples of shellfish were prepared for analysis immediately upon arrival in the laboratory or frozen until analysis. The shellfish were cleaned with fresh water. Each specimen was opened by cutting the adductor muscles, sand or other foreign material was removed and the meat transferred from the shell to a strainer to drain for several minutes. The strained meat was homogenized by blender or Ultraturrax, and a weight of approximately 100 g was used for analysis.

#### 3.2.2. Extraction Procedure

For the first extraction of lipophilic compounds (no yessotoxin) 300 mL acetone was added and the mixture homogenized (ultraturrax) for 2 minutes. The extracts were filtered, then the filtrate was collected and the solvent removed by rotary evaporation. The second extraction for yessotoxin was performed with methanol. The residue, after evaporation, was transferred with 30 mL dichloromethane and 60 mL of 60% methanol (in water) for liquid/liquid partition into a separation funnel.

After separation of these two phases, double extraction of dichloromethane phase was done with 60 mL portions of 60% methanol. All methanol phases were collected into the same glass vessel.

For determination of OA, DTXs, PTXs and AZAs, the dichloromethane solution was evaporated and residues were resolved by adding 4 mL of 1% Tween 60 solution. The prepared solution was used for mouse bioassay. Three mice weighing 18–22 g were injected with 1 mL of the solution. The death of two out of three mice within 24 hours is interpreted as the presence of one or more mentioned toxins at levels above those established in the Regulation (EC) No 853/2004 [27].

For determination of YTXs, the 16 mL of methanol extracts was evaporated. Four mL of 1% Tween 60 solution was added to the residues. The prepared solution was used for mouse bioassay. The death of two out of three mice within 6 hours is interpreted as the presence of YTX at levels above those established in the Regulation (EC) No 853/2004 [25].

### 3.3. Enzyme-Linked Immunosorbent Assay Method (ELISA) for the Determination of Yessotoxin (Ytx)

Shellfish tissue (2 g) was homogenized, using a Polytron homogeniser (2 min, 10,000 rpm), in 18 mL 90% methanol. The homogenate was centrifuged at 3,000 rpm for 10 minutes at room temperature. The supernatant was retained for analysis.

The assay was calibrated using a standard solution of YTX supplied in the ELISA kit from Biosense laboratories (Bergen, Norway).

### 3.4. Liquid Chromatography-Mass Spectrometry (LC-MS/MS)

Further investigations on those samples that gave positive results by the MBA for lipophilic toxins were carried out by liquid chromatography tandem mass spectrometry. Homogenized mussel tissues were extracted once with methanol 90% (sample to solvent ratio = 10). Extracts were then washed with n-hexane and filtered before injection into the LC. LC-MS/MS analyses were performed using a 1200 L triple quadrupole mass spectrometer equipped with an electrospray ionization source (Varian Inc., Walnut Creek, CA, USA).

#### 3.4.1. Chemicals and reagents

Methanol of HPLC grade was purchased from VWR (Milan, Italy). Water was distilled and passed through a MilliQ water purification system (Millipore Ltd., Bedford, MA, USA). Formic acid (reagent grade ≥ 95%) was purchased from Sigma-Aldrich (Steinheim, Germany), while ammonium hydrate for analysis was purchased from Carlo Erba (Milan, Italy). Certified reference materials for OA, DTX-1, PTX-2, YTX, GYM and SPX-1 were purchased from CNRC (Halifax, Canada) and were used for correct identification and quantification of toxins in mussel samples. Analogs of the previously mentioned toxins were identified by direct comparison with contaminated samples of known composition.

Chromatographic separation was performed using a 5 μm SunFire C18, 150 × 2.1 mm column (Waters Corporation, Milford, MA, USA) kept at 30 °C. Mobile phase A consisted of methanol:water (13:87, v/v) containing 50 mM formic acid and 4 mM ammonium hydrate. Mobile phase B consisted of methanol:water (90:10, v/v) containing 4.5 mM formic acid and 5.4 mM ammonium hydrate. Flow rate was 200 μL min^−1^. A step gradient elution was used: 0% B for 3 min, 0–97% B for 4 min and 97% B for 15 min. Re-equilibration time at the initial conditions was 8 min. Multiple reaction monitoring (MRM) experiments were carried out in positive/negative switching ion mode in order to investigate the presence of the following toxins: GYM (*m/z* 508 > 490 ES+); SPX-1 (*m/z* 692 > 444; *m/z* 692 > 164 ES+); OA and DTX-2 (*m/z* 803 > 255 ES−); DTX-1 (*m/z* 817 > 255 ES−); PTX-2 (*m/z* 876 > 823 ES+); PTX-1 (*m/z* 892 > 839 ES+); PTX-2-SA (*m/z* 894 > 823 ES+); PTX-6 (*m/z* 906 > 853 ES+); YTX (*m/z* 1141 > 1061 ES−); homoYTX (*m/z* 1155 > 1075 ES−); 45-OHYTX (*m/z* 1157 > 1077 ES−); 45-OHhomoYTX (*m/z* 1171 > 1091 ES−).

#### 3.4.2. Alkaline Hydrolysis

The hydrolysis procedure, as proposed by Mountfort *et al*. (2001) with slight modifications, was carried out by adding 125 μL of 2.5 M NaOH (100% aqueous) to 1 mL of sample crude extract. The mixture was kept at 76 °C for 40 min. After cooling to room temperature, the extract was acidified with 110 μL of 2.5 M HCl. The pH values of each extract after hydrolysis and addition of HCl ranged between 5.0 and 7.0, depending on the matrix.

### 3.5. Phytoplankton Analysis

Phytoplankton community structure was determined according to Utermöhl [28]. Phytoplankton samples were collected by both net and vertical PVC sampler. The net (upper diameter 29 cm, 20 μm mesh size) was towed vertically from the bottom to the surface. The results were expressed as N cells m^−2^. Samples collected by vertical PVC sampler were expressed as N cells L^−1^.

## 4. Conclusions

This report confirms YTXs, in addition to OA, as the main compounds responsible for the toxicity of lipophilic extracts of mussels (*M. galloprovincialis*) from Croatian waters. For the first time, this work highlights the YTX profile and reveals carboxy YTX as a major analog present in the examined mussels. At the same time, the concentration of YTXs producers in seawater is relatively low, which suggests that such toxins had accumulated in mussels in the period prior to collection. This is supported by the evidence that the oxidation products of YTX/homo YTX in mussels (carboxy YTX and its homo derivatives) are present in higher amounts than YTX/homo YTX themselves. Results obtained by three different methods (MBA, ELISA and LC-MS/MS) indicate that the ELISA method is the most sensitive for YTXs.

## Figures and Tables

**Table 1:** Dates and stations with positive mouse bioassay (MBA) for DSP in 2007 using Yasumoto’s method [13] and YTXs concentrations by ELISA.

Date	Station	MBA for DSP	YTXs by ELISA (μg kg^−1^)

24. April	SB 3	Positive	939
24. May	IP 3	Positive	1894
04. June	SB 2	Positive	n.a
27. November	MSB 5	Positive	n.a.

n.a. not analyzed.

**Table 2:** Dates and stations with positive mouse bioassay for DSP and YTX in the period from 20 August to 22 October 2007 using both Yasumoto’s method [13] and a modified version of Yasumoto’s method, which discriminated YTX from others DSP [14], and YTXs concentrations by ELISA).

Date	Station	MBA for DSP [13]	MBA for YTX [14]	YTXs by ELISA (μg kg^−1^)

20. August	MB 1	Negative	Positive	7900
10. September	MSB 4	Negative	Positive	42
17. September	IP 2	Negative	Positive	194
02. October	IP 2	Negative	Positive	567
02. October	LB 1	Positive	Negative	105
22. October	LB 1	Positive	Negative	n.a.

n.a. not analyzed.

**Table 3:** Lipophilic toxins’ levels detected by LC-MS/MS (μg kg^−1^) determined in positive mouse bioassay samples.

Station	date	YTX	Homo YTX	45- OH- YTX	45- OH- homo	Carboxy -YTX	Carboxy-homo YTX	PTX-2	PTXs	PTX2-SA	7-epi- PTX2-SA	OA	Total OA	AZAs
SB 3	24.04	131		89		traces	traces	traces	<25	traces	traces			<38
IP 3	24.05.	traces	72					traces	<25		traces			<38
MB1	20.08.		244		59	144	794	traces	<25	traces	traces		26	<38
MSB 4	10.09.					traces			<25	traces	traces			<38
IP 2	17.09							traces	<25					<38
IP2	02.10.		77					traces	<25	traces				<38
LB 1	02.10.		72					traces	<25	32	traces	341	787	<38
LB 1	22.10.							traces	<25	38	13	462	1041	<38
MSB 5	27.11.							traces	<25	27		37	92	<38
